# Supplementary material for: What Type of Patients Did PARAGON-HF Select? Insights from a Real-World Prospective Cohort of Patients with Heart Failure and Preserved Ejection Fraction
Source: J Clin Med. 2020 Nov 15;9(11):3669. doi: 10.3390/jcm9113669 (PMC7697501; doi:10.3390/jcm9113669)
Supplement: Supplementary file 1 [file jcm-09-03669-s001.pdf]

SUPPLEMENTARY TABLES

Supplementary Table S1. Cardiac magnetic resonance imaging of the real-world HFpEF cohort according to eligibility for PARAGON-HF.

Parameter	Missed inclusion criteria (± fulfilled excl. criteria) (*Cohort 2*) n = 175	Real-world PARGON-HF (*Cohort 1*) n = 170	Fulfilled inclusion and met exclusion criteria (*Cohort 3*) n = 82	*p*-Value	Cohort 2–Cohort 1 *p*-Value	Cohort 1–Cohort 3 *p*-Value	Cohort 2–Cohort 3 *p*-Value
**Cardiac magnetic resonance imaging**	**n = 92**	**n = 86**	**n = 39**				
Left ventricular end-diastolic diameter — mm	46.5 ± 6.5	46.5 ± 5.7	47.9 ± 6.5	0.458	0.990	0.237	0.271
Left ventricular end-diastolic volume — mL	127.0 ± 46.8	124.1 ± 37.5	140.7 ± 36.5	0.112	0.658	**0.023**	0.105
Left ventricular ejection fraction — %	65.6 ± 9.8	64.6 ± 10.0	63.1 ± 9.0	0.465	0.670	0.464	0.279
Left ventricular stroke volume — mL	80.1 ± 24.9	75.0 ± 26.7	85.3 ± 26.5	0.114	0.198	0.050	0.292
Left ventricular cardiac output — L/min	5.27 ± 1.72	5.03 ± 1.48	5.80 ± 1.91	0.068	0.335	**0.018**	0.132
Left ventricular mass — g	110.6 ± 32.8	109.3 ± 35.1	124.4 ± 34.2	0.123	0.814	0.057	0.064
Interventricular septum — mm	11.1 ± 2.1	11.1 ± 2.7	11.1 ± 1.9	0.992	0.906	0.975	0.944
Left atrial length — mm	64.5 ± 9.4	66.3 ± 8.9	68.1 ± 10.2	0.122	0.206	0.336	0.062
Left atrial area — cm^2	30.4 ± 9.0	30.9 ± 8.3	35.4 ± 11.4	**0.015**	0.699	**0.015**	**0.009**

Right ventricular end-diastolic diameter — mm	40.0 ± 7.1	40.2 ± 7.0	42.3 ± 8.2	0.245	0.869	0.151	0.118
Right ventricular end-diastolic volume — mL	141.1 ± 41.7	150.8 ± 52.4	183.9 ± 70.3	**<0.001**	0.869	**0.004**	**0.001**
Right ventricular ejection fraction — %	54.8 ± 10.3	50.5 ± 10.5	50.2 ± 11.7	**0.012**	**0.006**	0.911	**0.027**
Right ventricular stroke volume — mL	76.7 ± 20.4	74.6 ± 26.0	91.6 ± 33.4	**0.002**	0.544	**0.003**	**0.014**
Right ventricular cardiac output — L/min	5.07 ± 1.40	4.87 ± 1.58	6.10 ± 2.12	**0.001**	0.383	**0.001**	**0.008**
Right atrial length — mm	63.3 ± 8.4	66.3 ± 9.2	66.7 ± 10.2	**0.047**	**0.026**	0.827	0.052
Right atrial area — cm^2	27.4 ± 8.7	30.5 ± 10.0	31.1 ± 8.6	**0.036**	**0.029**	0.758	**0.028**
Pulmonary artery diameter — mm	28.5 ± 5.5	29.7 ± 5.3	32.3 ± 8.3	**0.006**	0.144	**0.038**	**0.003**
Extracellular volume — %	29.2 ± 3.9	30.1 ± 5.2	30.0 ± 4.5	0.619	0.357	0.980	0.484

Values are given as mean ± standard deviation. Bold numbers indicate statistical significance with p-values <0.05. Cardiac magnetic resonance imaging was performed in 50.8% of real-world HFpEF patients.

SUPPLEMENTARY FIGURES

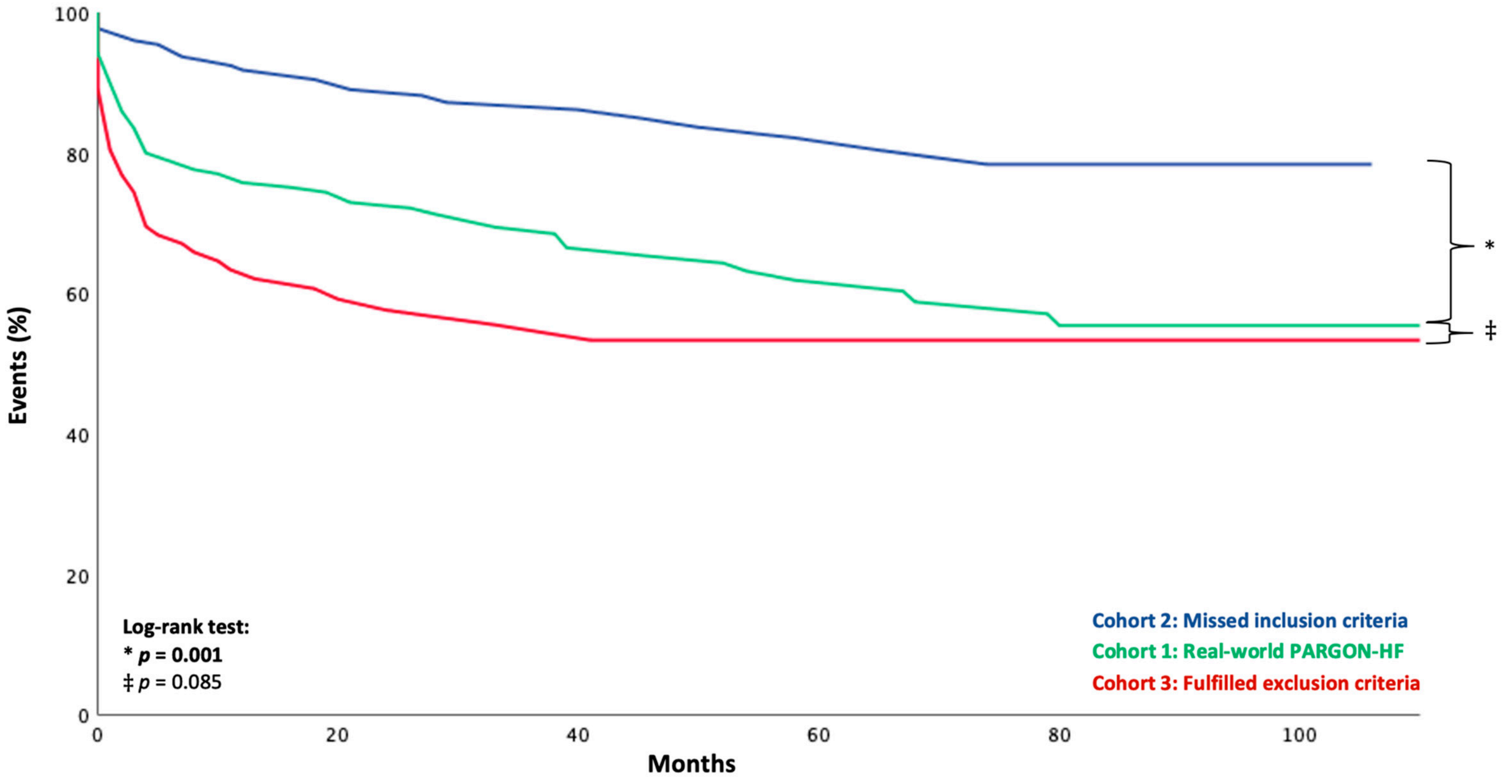

Supplementary Figure S1. Kaplan-Meier plots for hospitalization for heart failure according to eligibility for PARAGON-HF.

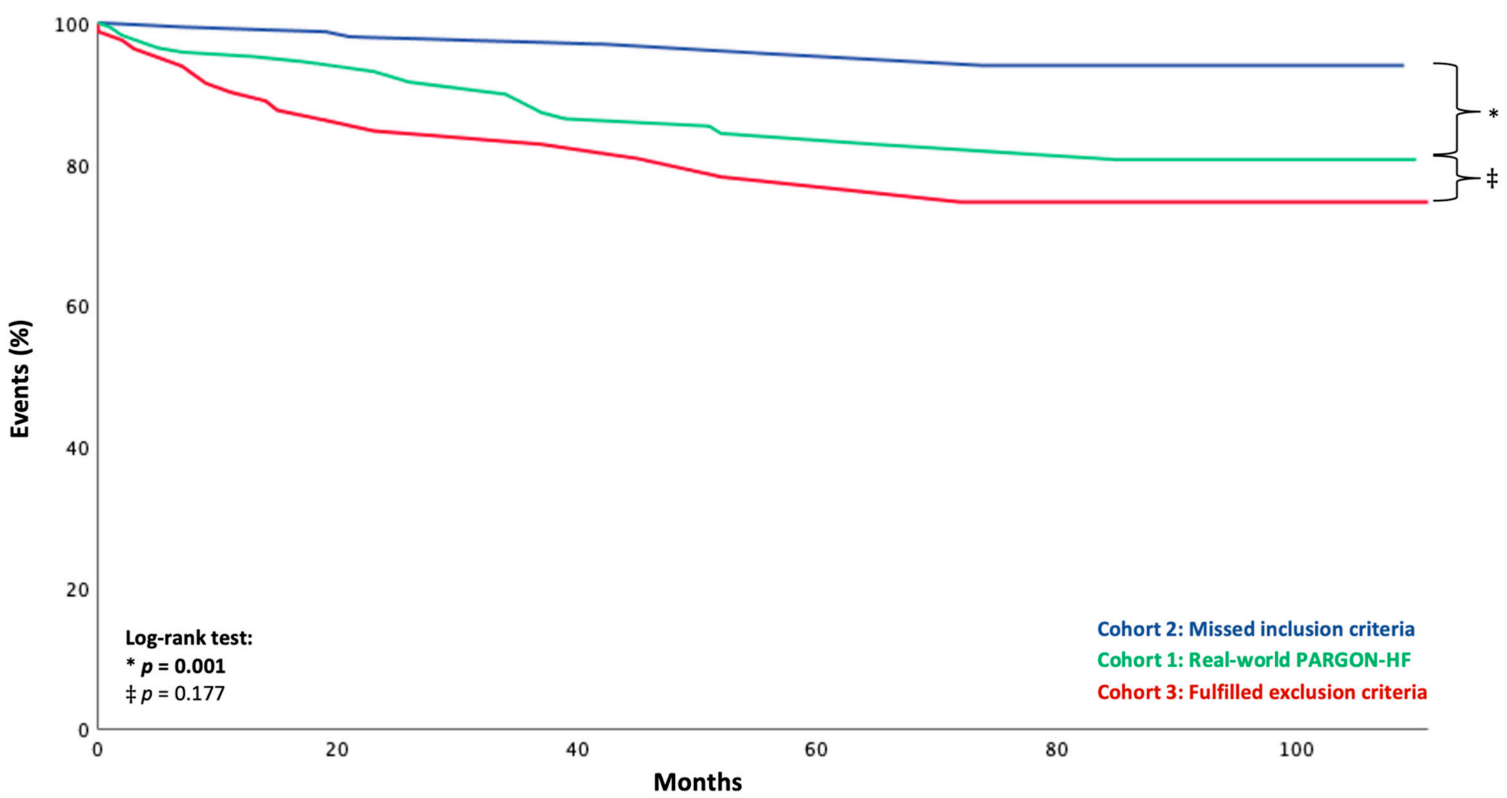

Supplementary Figure S2. Kaplan-Meier plots for death from cardiac causes according to eligibility for PARAGON-HF.

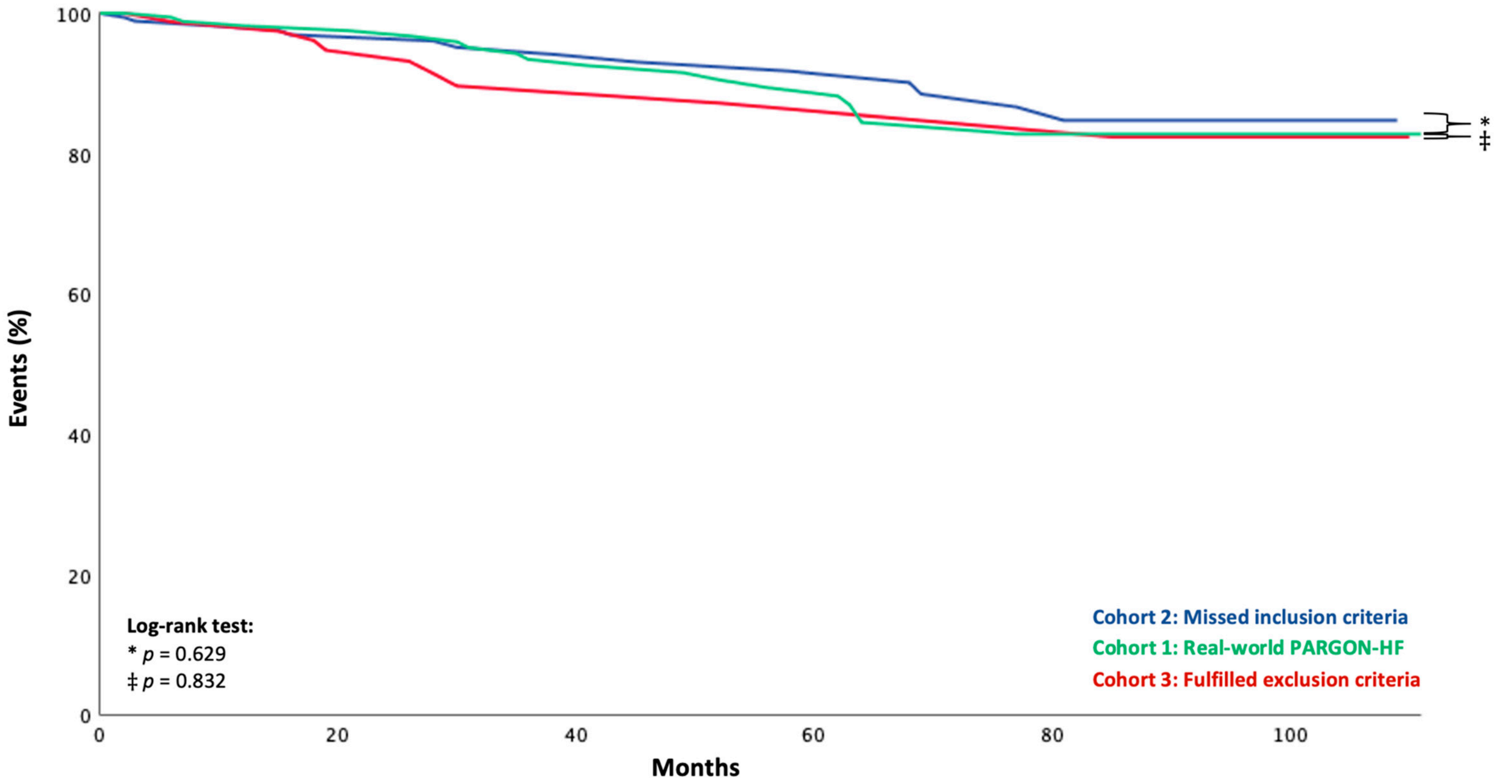

Supplementary Figure S3. Kaplan-Meier plots for death from non-cardiac causes according to eligibility for PARAGON-HF.

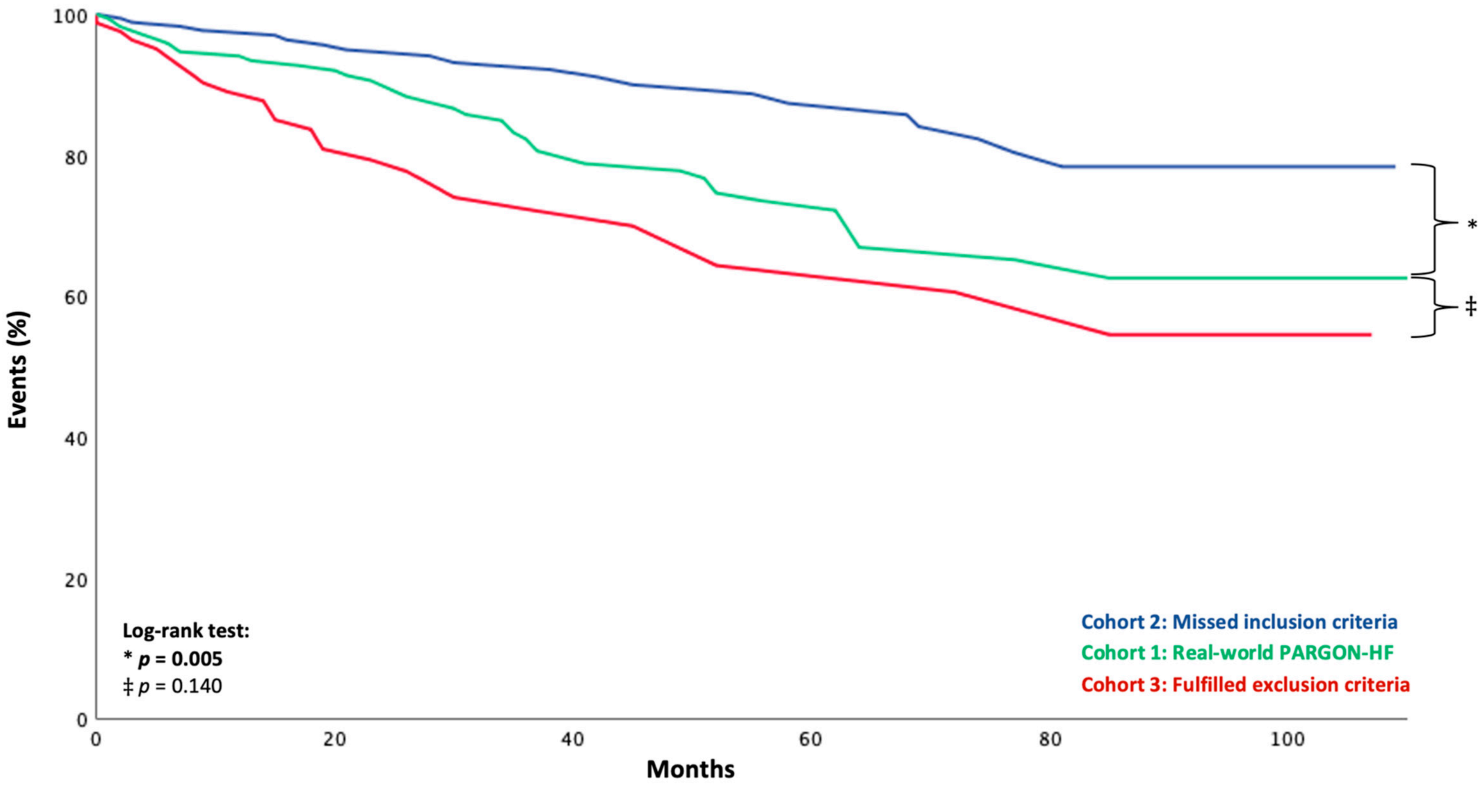

Supplementary Figure S4. Kaplan-Meier plots for death from any causes according to eligibility for PARAGON-HF.
